# Craniotomy, with the Implantation of Egg-Shell Membrane, for Jacksonian Epilepsy

**Published:** 1901-03

**Authors:** William Jones Greer


### CRANIOTOMY, WITH THE IMPLANTATION OF EGG-SHELL MEMBRANE, FOR JACKSONIAN EPILEPSY.

BY

William Jones Greer, F.R.C.S. Irel.

---

The introduction of egg-shell membrane by Freeman,[1] of Denver, for the prevention of adhesions between the brain and its coverings and the scalp appears to offer distinct advantages over the inversion method of Keen, the gold-foil of Beach, or the rubber tissue of Abbe. Freeman claims "that it is . . . easily obtained," that "it is not in the full sense of the term a 'foreign body,' but seems in a measure to incorporate itself with surrounding tissues without causing perceptible irritation or the formation of noticeable cicatricial deposits;" that it will not require a second operation for its removal. In an experiment on a dog, two months after the implantation of the membrane a microscopic section showed that the membrane had been replaced by a layer of adipose tissue permeated by blood-vessels.

The subject of this note was a young married lady, who, some eleven years ago, sustained a compound fracture of the skull, caused by the accidental falling of a roof-tile. She was very seriously ill after the accident, being unconscious for a period, with the frequent development of convulsive seizures. Several pieces of necrosed bone were removed, and the wound, which became septic, required almost two years for complete healing. The fits which were frequent during the first year of her illness became gradually less frequent, and for nearly nine years she was entirely free from them, but she has been a sufferer from severe headaches. Within the recent two years the fits have again appeared, and are gradually increasing in severity and regularity. For this she sought advice. She had gained considerably in general health by treatment, but the convulsive seizures were very little, if at all, controlled. The character of the fits as gleaned from the patient and her friends was, that for a day or two before the attack came on she felt depressed and out of sorts. About ten minutes before the onset she had very distinct muscular twitchings, commencing in the right thumb and gradually passing from group to group of muscles up the arm; the right foot and leg were occasionally similarly affected. The convulsions were always confined to the right side; there was no initial cry, and the attack was always followed by some loss of power in the arm and leg. She was usually unconscious at the height of the attack, and remained so for a variable period. The next day

[1] *Ann. Surg.*, 1898, xxviii. 455.

she habitually felt a very great deal better in health; latterly these seizures have been as frequent as one or two a week. Such was her particular history when I first saw her, about twelve months ago. She was then about twenty-two years of age, somewhat ill-nourished and anæmic. An examination of the various organs did not reveal anything of special interest. The recital of these symptoms, and the account of the accident, naturally attracted one's attention to the head, where a deep depression was to be seen, situated precisely over the upper end of the left fissure of Rolando, covering the upper portions of the ascending parietal and frontal convolutions. This depression was irregular, its longest axis being about two inches, its shortest one inch; its inner margin touched the longitudinal fissure. The bottom of the depression was occupied by a dense devious cicatrix firmly adherent to the structure underneath, the whole cavity being exceedingly tender to the touch. The diagnosis was obviously traumatic epilepsy; its slow development pointed forcibly to the irritation in the motor area being due to a tumour, a cyst, or most likely to the inclusion of brain tissue in the cicatrix. The treatment suggested to the patient was that under the circumstances an operation might possibly be expected to afford relief, it being at the same time explained that while the operation results are not always successful in conditions of the kind, the risk to life was not great, and the cause of the trouble was very apparent and afforded a reasonable hope of removal

On 23rd August, 1900, the head having been shaved and otherwise carefully prepared, I raised a large semilunar flap of scalp downwards from the left motor area. It was so strongly fixed to the stoma in the skull that I had some difficulty to avoid button-holing it. The skull opening was then seen to be filled with hard dense tissue, which was cut away with considerable trouble. During this part of the procedure very furious bleeding occurred, which for the moment caused me to think that I had opened the longitudinal sinus. When the hemorrhage was stopped, the brain could readily be seen pulsating very feebly, dark in appearance, soft and friable to the touch, with numerous fibrous septa coming up to join the removed cicatrix. These septa, with, roughly, two drachms of softened brain matter, were removed, and the cavity dried out. No projecting spiculæ of bone could be detected. After this, about a square inch of egg-shell membrane was placed between the brain and the skull opening; another portion of membrane was placed outside over the opening, and the scalp flap brought into position over it, sutured with silkworm gut, and a sterile dressing applied. The wound had an aseptic after-course, and healed *per primam*.

To sum up the salient features of the case, it presents itself as traumatic epilepsy due to a compound fracture of the skull, and the subsequent formation of dense adhesions between brain, membrane, and scalp. The treatment adopted was, opening of the dura, which, according to Kocher,[1] has a curative effect *per se*, possibly by lowering tension, the removal of the diseased motor area in the manner of Von Bergmann, and the prevention of adhesions by the insertion of egg-membrane as suggested by Freeman.

[1] *Ann. Surg.*, 1900, xxxi. 637.

The more remote history of the case exhibits details of some interest, at the moment of writing, six months after the operation. I have to record that about two months ago, in consequence of a slight injury, the old cicatrix broke down and left a small opening in the scalp, nearly a quarter of an inch in extent, through which could easily be seen the second portion of egg-shell membrane, white and glistening; fearing that, by the time this portion would be surrounded by granulations, the old scar might have entirely given way, as it would have very little vascular support until these granulations carried an abundance of life to it, with fine forceps I removed this second piece of membrane; it came out in its entirety; it was still tough, and stood well the strain of being dragged out, its edges being, apparently, held by granulation tissue. Macroscopically there was no alteration in its structure. Underneath it was a layer of granulations; nothing of the first piece of egg-membrane was to be seen. The small scalp opening soon filled up. Thus the construction of the breach, I take it, is that next to the brain is the first piece of egg-shell membrane, after that dense fibrous tissue, then scalp.

I feel that it was an undoubted mistake to put in a second portion of membrane, and on another occasion I shall be quite satisfied with one patch.

Up to the present, appearances are in favour of a good result; the old cicatrix is not in the least tender. It is supple and freely movable, and there has been no recurrence of the fits.

